# The mediating roles of the challenge appraisal in the relationship between the coach–athlete relationship and adolescent athletes' burnout

**DOI:** 10.3389/fpsyg.2026.1770500

**Published:** 2026-06-18

**Authors:** Sun-Ho Lee, Hunhyuk Choi

**Affiliations:** 1 Department of Social Physical Education, Sunchon National University, Suncheon, Republic of Korea; 2 Department of Physical Education, Kangwon National University, Chuncheon, Republic of Korea

**Keywords:** adolescent athletes, athlete burnout, challenge appraisal, coach–athlete relationship, mediation effect

## Abstract

**Purpose:**

This study examined the relationships among the coachathlete relationship, challenge appraisal, and athlete burnout in adolescent athletes, with a focus on the mediating role of challenge appraisal.

**Design/methodology/approach:**

A total of 323 adolescent athletes (231 males, 92 females) participated in the study. Data were analyzed using SPSS 26.0 and AMOS 26.0, and the mediating effects were examined using the bootstrapping method.

**Results:**

First, the coachathlete relationship had a significant positive effect on challenge appraisal (β = 0.374, *p* < 0.001). Second, challenge appraisal negatively influenced athlete burnout (β = −0.173, *p* < 0.01). Third, the coachathlete relationship had a significant negative effect on athlete burnout (β = −0.210, *p* < 0.01). Fourth, challenge appraisal partially mediated the relationship between the coachathlete relationship and athlete burnout (indirect effect = −0.079, *p* < 0.01).

**Conclusion:**

These findings provide theoretical insights and practical implications for designing psychological intervention programs aimed at preventing burnout in adolescent athletes. The study also emphasizes the importance of coaching strategies and coach education programs that foster emotionally supportive and motivational environments in sports.

## 1 Introduction

In adolescent sports environments, there is a growing recognition that technical and physical development must be accompanied by psychological and interpersonal support structures to promote athletes' performance and long-term participation in sport (Jowett, 2017). Among these psychosocial concerns, athlete burnout has emerged as a major issue, often leading to dropout, negative emotional reactions, and impaired well-being (Pacewicz et al., 2020). Burnout can cause serious emotional and physical consequences for young athletes, underscoring the need to identify factors that can buffer against its development (Gustafsson et al., 2011). Therefore, understanding how the coach–athlete relationship and athletes' perceptions of sport-related tasks—particularly their appraisal of challenge—operate to reduce or exacerbate burnout is essential for fostering healthy adolescent sport environments.

Coaches play a central role in shaping athletes' growth during training sessions, competitions, and broader sport-related contexts (Nicol and Allen, 2024). In this regard, maintaining a positive coach–athlete relationship is widely considered a core aspect of effective coaching and is closely linked to both skill development and performance outcomes (Bora, 2025). Much of the contemporary research in this area has been informed by Jowett's (2025)) interdependence model, also known as the 3 + 1Cs framework, which conceptualizes high-quality dyadic relationships through closeness, commitment, and complementarity. Empirical and meta-analytic findings consistently show that supportive and well-functioning coach–athlete relationships—characterized by trust, respect, and reciprocal interaction—serve as strong protective factors against athlete burnout (Bora, 2025; Jowett and Shanmugam, 2016). Such relationships satisfy athletes' basic psychological needs, assist with stress regulation, reduce emotional exhaustion, and promote resilience and sustained motivation. In contrast, poor communication, relational conflict, or controlling coaching behaviors undermine athletes' autonomy and emotional stability, thereby heightening vulnerability to burnout (Fan et al., 2023).

Beyond relational influences, athletes' cognitive evaluations of sport-related demands—such as their appraisal of challenge—also shape how they respond to competitive pressures (Nicholls et al., 2012). According to the stress-coping framework (Folkman and Lazarus, 1985; Kerdijk et al., 2016), athletes who perceive sport tasks as meaningful and manageable are more likely to engage with motivation and persistence, whereas those who interpret demands as overwhelming or threatening may experience heightened stress and reduced confidence. Supportive coaching environments and high-quality interpersonal relationships have been shown to facilitate more adaptive challenge appraisal, encouraging athletes to view demanding situations as opportunities for development rather than as sources of threat (Meijen et al., 2020).

Recent research on adolescent athletes has highlighted a wide range of factors associated with burnout, including motivation, psychological skills, training environments, and interpersonal relationships (da Silva et al., 2025; Gerber et al., 2024; Isoard-Gautheur et al., 2016). Collectively, these studies indicate that burnout results from a complex interplay of environmental, psychological, and sociocultural influences. Madigan et al. (2022)) pointed out that adolescent athletes today report symptoms of burnout more frequently than in the past, identifying early specialization, limited coping resources, increased external pressure, and threats to competence as major contributing factors. In contrast, West et al. (2016)) reported that autonomy-supportive coaching, positive team climates, and high-quality interpersonal relationships function as factors that mitigate these burnout risks. These environments enhance psychological well-being and offer resilience against the psychological demands of competitive sport (Eklund and DeFreese, 2020; Gustafsson et al., 2017).

Despite this progress, relatively little empirical research has examined how the coach–athlete relationship and athletes' challenge appraisal jointly contribute to burnout among adolescent athletes. Clarifying the mechanisms through which relational and cognitive factors shape burnout will contribute to a more comprehensive understanding of the processes underlying young athletes' psychological well-being (Kim et al., 2025). More importantly, identifying how supportive coach–athlete relationships enhance challenge appraisal and reduce burnout can offer practical guidance for building healthier, more sustainable coaching practices in adolescent sport settings.

## 2 Literature review

### 2.1 Coach-athlete relationship, challenge appraisal, and athlete burnout

Athlete burnout is a chronic state of psychological exhaustion caused by repetitive emotional and physical stress. In the context of sports, burnout can be understood through three dimensions: emotional exhaustion, sport devaluation, and a sense of reduced personal accomplishment (Raedeke and Smith, 2001). This persistent psychological exhaustion may eventually lead to athletes disengaging from sports and dropping out. Adolescent athletes are particularly vulnerable to burnout due to multiple pressures, such as balancing sports training with academic studies, parental and coaching expectations, and peer competition (Gustafsson et al., 2011, 2023). In light of these risks, identifying key social factors that serve to prevent or mitigate burnout symptoms is critical. Among these, the coach–athlete relationship has emerged as a primary social determinant that significantly shapes an athlete's psychological resilience.

Coaches play a central role in shaping athletes' development and well-being. They are not simply instructors of sports skills but also serve as role models and providers of emotional support. Positive coach–athlete relationships have been identified as a critical factor in promoting psychological stability, intrinsic motivation, and sustained engagement in sports (Jowett and Cockerill, 2003; Kim and Choi, 2024). High-quality coach–athlete relationship characterized by closeness, commitment, and complementarity have been associated with increased intrinsic motivation, psychological well-being, and reduced burnout (Lorimer and Jowett, 2010; Mageau and Vallerand, 2003).

Beyond providing emotional support, this relational quality functions as a psychological foundation that shapes how athletes cognitively evaluate competitive demands, specifically influencing their challenge appraisal. Challenge appraisal is another important factor that influences athletes' psychological experiences. When athletes perceive sports tasks as challenging opportunities rather than burdens, they are more likely to experience flow—a state in which their skills and task difficulty are optimally balanced—maximizing psychological satisfaction and motivation (Csikszentmihalyi, 1990). Flow experiences can reduce emotional exhaustion, strengthen self-efficacy, and support prolonged engagement in sports. Self-determination theory suggests that environments that respect autonomy and competence contribute to lower burnout levels, even under stress (Deci and Ryan, 2000).

Theoretical frameworks further indicate that the coach–athlete relationship and challenge appraisal are interconnected in influencing athlete burnout (Nicholls, 2021). Supportive coach–athlete interactions can promote positive challenge appraisal, which in turn enhances motivation, flow, and resilience, thereby reducing the risk of burnout (Jowett and Ntoumanis, 2004; Nicholls and Perry, 2016). Conversely, negative relationships with coaches or unsupportive interactions can lead athletes to perceive stress as threatening, reduce their challenge appraisal, and increase vulnerability to burnout (Choi and Jung, 2020). Therefore, understanding the mediating roles of the coach–athlete relationship and challenge appraisal is essential for promoting the psychological well-being of adolescent athletes.

In Korea, the results-oriented coaching style remains the predominant approach in adolescent sports environments, where emotional abilities or relational skills are relatively neglected. Such an environment highlights the importance of cultivating positive coach–athlete relationships and fostering challenge appraisal as key psychological resources that can enhance athletes' competence, autonomy, and engagement. Coaches who recognize and develop these relational and cognitive aspects are more likely to support athletes' psychological well-being and reduce burnout risk. Given the severe impact of burnout on adolescent development, it is imperative to examine these interpersonal dynamics to provide a scientific basis for intervention.

### 2.2 Roles of coach–athlete relationship and challenge appraisal

In adolescent athletes, athlete burnout is influenced by multiple psychosocial mechanisms rather than emerging from a single direct cause. In particular, the quality of the coach–athlete relationship and athletes' cognitive evaluations of sport-related demands have been identified as key factors associated with burnout. Coaches play an essential role not only as instructors of sport skills but also as significant figures who shape athletes' psychological experiences within sport contexts. According to Jowett and Cockerill's (2003)) 3 + 1Cs model, the coach–athlete relationship is characterized by closeness, commitment, and complementarity, which collectively contribute to athletes' psychological stability and autonomous motivation. Previous studies have consistently shown that high-quality coach–athlete relationships help athletes interpret coaches' feedback as opportunities for growth rather than sources of threat, thereby reducing vulnerability to burnout (Kim and Choi, 2024; Lorimer and Jowett, 2010). In addition, autonomy-supportive coaching behaviors and positive interpersonal interactions have been found to enhance athletes' self-efficacy and identity, which serve as protective factors against burnout (Mageau and Vallerand, 2003).

Challenge appraisal represents an important cognitive mechanism through which athletes respond to sport-related stressors. When athletes perceive demanding situations as challenges rather than threats, they are more likely to experience intrinsic motivation, persistence, and adaptive coping. According to Csikszentmihalyi's (1990)) flow theory, challenge appraisal is a core condition for entering a state of flow, which occurs when perceived task difficulty is well matched with individual ability. Experiencing flow has been shown to buffer key components of burnout, such as emotional exhaustion and reduced self-efficacy, while promoting long-term engagement and a sense of accomplishment in sport. Furthermore, self-determination theory (Deci and Ryan, 2000) emphasizes that environments supporting autonomy and competence facilitate adaptive motivation and lower burnout, even under conditions of high stress.

From this perspective, the coach–athlete relationship may play an important role in shaping athletes' challenge appraisal by creating a supportive interpersonal climate that encourages positive interpretations of demanding sport situations. Athletes who perceive strong relational support from their coaches are more likely to maintain challenge-oriented appraisals during training and competition, which in turn reduces the likelihood of burnout. Previous research has demonstrated that positive coaching environments and constructive interpersonal relationships are associated with higher levels of challenge appraisal, flow experiences, and lower burnout among athletes (Jowett and Ntoumanis, 2004; Nicholls and Perry, 2016). Conversely, athletes exposed to poor relational climates or controlling coaching behaviors are more likely to appraise stressors as threats, leading to heightened emotional exhaustion and burnout (Choi and Cho, 2019). Integrating these perspectives, it can be argued that the coach–athlete relationship and challenge appraisal operate as interconnected psychological mechanisms in the prevention of burnout. By fostering a secure relational base, coaches may enable athletes to maintain a challenge-oriented mindset even under high pressure.

Hypothesis 1: The coach-athlete relationship will be positively predict athletes' challenge appraisal.

Hypothesis 2: Challenge appraisal will be negatively associated with athlete burnout.

Hypothesis 3: Challenge appraisal will mediate the relationship between the coach–athlete relationship and athlete burnout.

### 2.3 Significance of the present study for the Korean adolescent sports environment

The present study aims to examine the roles of the coach–athlete relationship and challenge appraisal in athlete burnout, offering important empirical implications for the Korean adolescent sports environment. Specifically, the present study proposed and tested a structural model that integrates relational and cognitive factors by examining the quality of the coach–athlete relationship and athletes' appraisal of sport-related demands in explaining burnout. By focusing on these psychological mechanisms, the study offers a theoretically grounded and practically applicable framework for understanding burnout in adolescent sport settings.

Empirically, a coaching culture that emphasizes performance outcomes and competitive success remains dominant within the Korean sports system. Within this environment, adolescent athletes are frequently exposed to high levels of psychological pressure and emotional burden, which increase their vulnerability to burnout (Gustafsson et al., 2011). Against this backdrop, the present study highlights the importance of relationship quality and cognitive appraisal processes as key protective factors against burnout, consistent with prior research emphasizing the role of supportive coaching relationships and adaptive cognitive appraisals in athlete well-being (Jowett and Ntoumanis, 2004; Mageau and Vallerand, 2003).

The findings suggest that supportive coach–athlete relationships contribute to athletes' psychological stability and motivation, while challenge-oriented appraisal enables athletes to interpret demanding training and competition situations in a more adaptive manner, thereby reducing burnout risk (Csikszentmihalyi, 1990; Deci and Ryan, 2000). From a practical perspective, these results underscore the need to move beyond a purely performance-oriented coaching approach toward coaching practices that emphasize relationship-building and positive psychological climates. By demonstrating how the coach–athlete relationship and challenge appraisal function to reduce burnout, this study provides meaningful guidance for coach education programs and the design of adolescent sport development initiatives. Furthermore, the findings offer a scientific foundation for the development of sport psychological intervention programs tailored to the Korean context. Ultimately, this study contributes to ongoing efforts to promote a healthier, more person-centered sports culture where emotional well-being, relationship-focused guidance, and the sustained development of intrinsic motivation among adolescent athletes.

## 3 Methods

### 3.1 Research model

**Figure 1:**
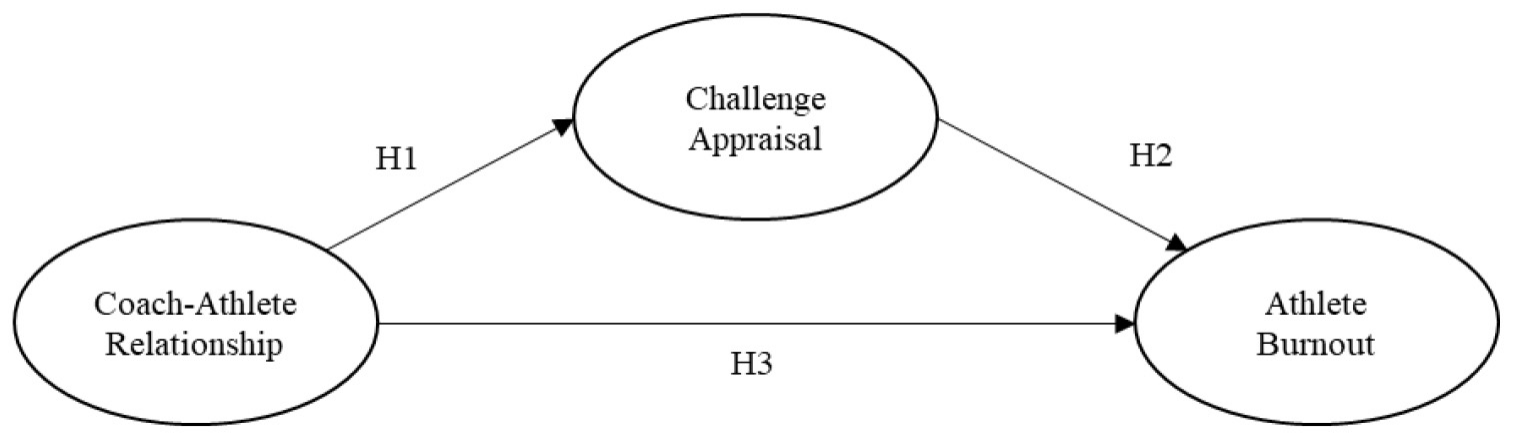
Conceptual research model.

This study proposes a conceptual research model to investigate the structural relationships among the coach–athlete relationship, challenge appraisal, and athlete burnout in adolescent athletes (see Figure 1). A cross-sectional research design was employed, and the proposed hypotheses were tested using Structural Equation Modeling (SEM). In this model, the coach–athlete relationship was specified as the independent variable, challenge appraisal as the mediating variable, and athlete burnout as the dependent variable. The model specifically examines the indirect mechanism through which the quality of the coach–athlete relationship influences athlete burnout via athletes' cognitive appraisal processes.

### 3.2 Participants

The population of this study comprised adolescent athletes in South Korea. Using a convenience sampling method, a total of 323 participants were recruited from middle and high schools located in Seoul, Gyeonggi, and Chungbuk provinces. Participants were included if they met the following criteria: (a) aged between 14 and 18 years, (b) official registration as a student-athlete with the Korea Sport and Olympic Committee or an affiliated school sports club, and (c) active engagement in regular training and competitive events at the time of recruitment. Conversely, participants were excluded if they: (a) were not currently active in organized sports, (b) had less than one year of formal competitive experience, or (c) submitted incomplete or unreliable survey responses.

The final sample consisted of 231 males (71.5%) and 92 females (28.5%) across 12 diverse sports disciplines, including shooting (*n* = 53, 16.4%), kendo (*n* = 46, 14.2%), wrestling (*n* = 37, 11.5%), baseball (*n* = 33, 10.2%), swimming (*n* = 26, 8.0%), basketball (*n* = 25, 7.7%), boxing (*n* = 23, 7.1%), badminton (*n* = 19, 5.9%), tennis (*n* = 18, 5.6%), fencing (*n* = 15, 4.6%), handball (*n* = 14, 4.3%), and taekwondo (*n* = 14, 4.3%). The mean age of the participants was 16.49 years (SD = 1.72), and the average duration of competitive participation was 3.56 years (SD = 2.58), confirming their status as active competitive athletes. Notably, 51.4% (*n* = 166) of the participants had received awards in competitions, and 56.0% (*n* = 181) reported experiencing at least one sports-related injury.

Although convenience sampling was adopted for reasons of accessibility, this method may limit the generalizability of the findings. Therefore, the results should be interpreted with caution, as the sample may not fully represent the broader population of adolescent athletes in South Korea. To mitigate this limitation, participants were recruited from multiple regions and a variety of sports disciplines to enhance sample diversity. Ethical approval and informed consent procedures are described in the Procedure section.

### 3.3 Measures

#### 3.3.1 Coach–athlete relationship

In this study, the coach–athlete relationship was assessed using the Korean version of the Coach–Athlete Relationship Questionnaire (CART-Q) developed by Jowett and Ntoumanis (2004)). The Korean-translated version of the CART-Q (KrCART-Q) for Korean athletes and coaches was developed and validated by Kim and Park (2008)). This scale consisted of 11 items organized into three subfactors: closeness (four items), commitment (three items), and complementarity (four items). In the confirmatory factor analysis, the estimated fit indices were as follows: *x*^2^ = 80.921, df = 30, *p* < 0.001, TLI = 0.978, CFI = 0.985, RMSEA = 0.070, and SRMR = 0.024, indicating a good fit. The Cronbach's α values for each subfactor were 0.947 for closeness, 0.802 for commitment, and 0.937 for complementarity. Each item is rated on a seven-point Likert scale ranging from one point (= “Not at all”) to seven points (= “Very much”). There were no reverse-coded items in this scale. Subscale scores were calculated by averaging the items within each factor, where higher scores indicate a more positive perception and stronger perception of the coach–athlete relationship.

#### 3.3.2 Challenge appraisal

The challenge appraisal was measured using a Korean-translated version of the Student Perceptions of Classroom Quality (SPOCQ) developed by Gentry and Owen (2004)). Lee and Chae (2016)) developed and validated the Korean-translated version of the SPOCQ. This scale consists of a single factor and seven items. Each item is rated on a five-point Likert scale ranging from one point (= Not at all) to five points (= Very much). Confirmatory factor analysis revealed the following fit indices: *x*^2^ = 35.711, df = 12, *p* < 0.001, TLI = 0.973, CFI = 0.985, RMSEA = 0.084, and SRMR = 0.025, indicating a good fit. The Cronbach's α value was 0.936, showing the reliability of the scale. This scale did not include any reverse-coded items. The total score was computed by averaging the responses to all items, with higher scores reflecting a stronger tendency to perceive sport-related demands as challenges.

#### 3.3.3 Athlete burnout

Athlete burnout was measured using a Korean-translated version of the Athlete Burnout Questionnaire (ABQ) developed by Raedeke and Smith (2001)) for use in sports situations. Choi et al. (2017)) developed and validated the Korean-translated version of the ABQ for Korean athletes. The ABQ consists of 15 items and three subfactors: emotional and physical exhaustion (five items), reduced sense of accomplishment (five items), and sport devaluation (five items). In the confirmatory factor analysis, two items (items one, two from ‘reduced sense of accomplishment') showed standardized regression weights of less than 0.50 and were thus deleted to ensure structural validity. Following the deletion, the fit indices indicated a good fit: *x*^2^ = 119.971, df = 49, *p* < 0.001, TLI = 0.952, CFI = 0.964, RMSEA = 0.072, and SRMR = 0.042. The Cronbach's α values for each subfactor were 0.767 for reduced sense of accomplishment, 0.889 for emotional and physical exhaustion, and 0.905 for sport devaluation. Although the original ABQ contains reverse-coded items in the “reduced sense of accomplishment” factor the items were used in their non-reversed form or recoded to ensure that higher scores consistently indicated higher levels of athlete burnout. Subscale overall scores were calculated averaging all retained items.

### 3.4 Measurement model specification

In this study, when specifying the measurement model, challenge appraisal was modeled using its original observed indicators. In contrast, item parceling was applied to the coach–athlete relationship and athlete burnout constructs based on the sub-factors identified in previous research (Kline, 2011). Specifically, the items within each subfactor were averaged to create representative indicators for each latent variable. This approach was adopted to enhance model parsimony.

### 3.5 Procedure

This study was conducted after the researchers received research ethics education (IRB-KNUE-202505-SB-0189-01) provided by the researcher's affiliated institution (Kangwon National University). The researcher personally contacted each university coach and sent them the content of the survey questionnaire via e-mail. After the coaches reviewed the questionnaire content and gave approval for the survey, the researcher and co-researchers personally visited each sports team and conducted the survey. The athletes voluntarily participated in the survey; the purpose and expected effects of the study were explained to them. In addition, the survey was conducted after the participants were informed that their personal information would not be disclosed. The participants were asked to respond sincerely to the questions when filling out the questionnaire. A small gift (a Starbucks gift certificate) was given to each participant as a token of appreciation.

### 3.6 Data analysis

Data analysis was performed using SPSS 26.0 and AMOS 26.0. First, data screening procedures were conducted to examine multivariate normality, outliers, and multicollinearity. Normality was assessed through skewness and kurtosis values, and the acceptability of these values was determined based on the thresholds suggested by Kline (2023)). Potential multivariate outliers were identified using Mahalanobis distance (*D*^2^). Multicollinearity was evaluated by reviewing the correlation coefficients among the variables, ensuring they did not exceed the recommended threshold (*r* < 0.80).

Second, following the two-step approach suggested by Anderson and Gerbing (1988)), the measurement model was first validated using Confirmatory Factor Analysis (CFA), followed by Structural Equation Modeling (SEM) to test the hypothesized relationships. To ensure the structural integrity and convergent validity of the measurement model, Average Variance Extracted (AVE) and Construct Reliability (CR) were calculated. Following the recommendations of Fornell and Larcker (1981)) and Hair et al. (2019)), the acceptable thresholds were set at AVE > 0.50 and CR > 0.70. During this process, items with low factor loadings (under 0.50) that compromised the model fit were removed to improve model parsimony. Specifically, two items from the ‘reduced sense of accomplishment' scale were excluded due to low factor loadings. Model fit was evaluated based on the criteria suggested by Hair et al. (2019)), utilizing several indices: the chi-square to degrees of freedom ratio (χ^2^/df ≤ 3), the Tucker–Lewis Index (TLI ≥ 0.90), the Comparative Fit Index (CFI ≥ 0.90), the Root Mean Square Error of Approximation (RMSEA ≤ 0.08), and the Standardized Root Mean Square Residual (SRMR ≤ 0.08). Finally, the mediating effect of challenge appraisal was examined using the bootstrapping method with 5,000 resamples. The significance of the indirect effect was determined using a 95% bias-corrected confidence interval (BC 95% CI); mediation was considered statistically significant when the confidence interval did not include zero (Preacher and Hayes, 2008). The statistical significance level was set at α = 0.05.

### 3.7 Testing for the convergent validity of measures

In this study, the coach-athlete relationship was specified as the exogenous variable, challenge appraisal as the mediating variable, and athlete burnout as the endogenous variable. Structural equation modeling was conducted to examine the relationships among these variables. The measurement model was tested before the analysis of the structural model. After verifying the measurement model, the structural model was established. The convergent validity of the variables of coach–athlete relationship, challenge appraisal, and athlete burnout was tested to verify the measurement model (Table 1). Specifically, the construct reliability (C.R.) values of each latent variable ranged from 0.782 to 0.949, exceeding the threshold (≥0.70), and the AVE (average variance extracted) values ranged from 0.557 to 0.825, exceeding the threshold (≥0.50). Hence, there was no problem with the convergent validity of the data (Fornell and Larcker, 1981).

**Table 1:** Standardized coefficients of the latent variables and verification of convergent validity and reliability in the measurement model.

Latent variables	Observable variables	Standardized coefficients	AVE	C.R.	Reliability
Coach-athlete relationship	Closeness	0.898	0.825	0.934	0.940
	Commitment	0.899			
	Complementarity	0.954			
Challenge appraisal	Challenge appraisal 1	0.740	0.727	0.949	0.936
	Challenge appraisal 2	0.840			
	Challenge appraisal 3	0.855			
	Challenge appraisal 4	0.915			
	Challenge appraisal 5	0.883			
	Challenge appraisal 6	0.736			
	Challenge appraisal 7	0.762			
Athlete burnout	Reduced sense of accomplishment	0.600	0.557	0.782	0.761
	Emotional/physical exhaustion	0.608			
	Sport devaluation	0.948			

## 4 Results

### 4.1 Preliminary analysis

As the first step of the analysis, descriptive statistics and correlations among the study variables were examined (Table 2). The normality assumption of the data was evaluated using skewness and kurtosis statistics. To determine the acceptability of these values, we applied the thresholds suggested by Kline (2023)), which consider absolute values of skewness under 3.0 and kurtosis under 10.0 as evidence of a normal distribution. As shown in Table 2, all observed variables fell within these recommended ranges, confirming that the data was suitable for structural equation modeling (SEM) analysis using maximum likelihood estimation. Specifically, the coach–athlete relationship showed the highest mean score (*M* = 6.13), followed by challenge appraisal (*M* = 3.79), and athlete burnout (*M* = 2.61).

**Table 2:** Descriptive statistics and correlation analysis.

Latent variables	M	SD	Skewness	Kurtosis	1	2	3
1. Coach–athlete Relationship	6.13	1.00	−1.357	1.233	1		
2. Challenge appraisal	3.79	0.74	0.333	−1.041	0.370^**^ (0.139)	1	
3. Athlete Burnout	2.61	0.79	0.605	0.993	–0.184^**^ (0.033)	–0.173^**^ (0.029)	1

^**^p < 0.01

The numbers in parentheses represent squared correlation coefficients.

Correlation analysis confirmed that the coach-athlete relationship was positively associated with challenge appraisal (*r* = 0.370, *p* < 0.01), and negatively with athlete burnout (*r* = −0.184, *p* < 0.01). Challenge appraisal also showed a significant negative correlation with athlete burnout (*r* = −0.173, *p* < 0.01). Discriminant validity was secured as the squared correlation coefficients between constructs were lower than the Average Variance Extracted (AVE) values reported in the subsequent structural model analysis.

### 4.2 Structural model and hypothesis testing

**Figure 2:**
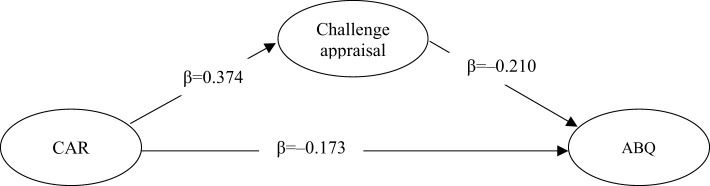
Structural equation modeling of the relationship between coach-athlete relationship, challenge appraisal and athlete burnout.

The structural model's fit was evaluated before testing the hypotheses. To ensure a concise and consistent evaluation, the model fit indices and validity measures (AVE and CR) are integrated into this stage. The results indicated a good fit to the data: *x*^2^ = 141.394, df = 61, TLI = 0.961, CFI = 0.969, RMSEA = 0.069, and SRMR = 0.047. All AVE values exceeded the recommended threshold of 0.05, and construct reliability (CR) values were above 0.70, ensuring sufficient convergent validity within the structural framework. The path coefficients for the hypothesis testing are presented in Table 3, Figure 2. The coach–athlete relationship had a significant positive effect on challenge appraisal (β = 0.374, *p* < 0.001). Furthermore, the coach–athlete relationship had a significant negative effect on athlete burnout (β = −0.210, *p* < 0.01), and challenge appraisal also had a significantly and negatively influenced athlete burnout (β = −0.173, *p* < 0.01).

**Table 3:** SRW, direct, indirect, and total effects in the structural model.

Path	SRW	C.R.	Direct effects	Indirect effects	Total effects
CAR → CA	0.374	5.930	0.275	–	0.275^***^
CA → ABQ	−0.173	−2.616	−0.285	–	−0.285^**^
CAR → ABQ	−0.210	−3.121	−0.173	−0.079	−0.252^**^

^**^p < 0.01.

^***^p < 0.001.

To verify the significance of the indirect effect, bootstrapping was performed as described in the methods section. The analysis indicated that the indirect effect of the coach–athlete relationship on athlete burnout through challenge appraisal was statistically significant (β = −0.079, *p* < 0.05), with a 95% bias-corrected confidence interval [−0.148, −0.016] that did not include zero. These results confirm that challenge appraisal significantly mediated the relationship between the coach–athlete relationship and athlete burnout (Table 4).

**Table 4:** Estimation of the mediation effects.

Indirect effects	Bootstrap standard errors	Sig	Bootstrapping	
		BC 95% CI lower bounds	Upper bounds
−0.079	0.033	0.017^*^	−0.148	−0.016

^*^p < 0.05.

## 5 Discussion

This study aimed to examine the mediating role of challenge appraisal in the relationship between the coach-athlete relationship and athlete burnout among adolescent athletes. The findings supported a partial mediation model, indicating that the coach-athlete relationship and challenge appraisal were significant factors in mitigating athlete burnout. The key findings of this study are discussed in detail below.

First, the results of this study showed that the coach–athlete relationship had a significant positive effect on athletes' challenge appraisal. This indicates that positive interactions, trust, and emotional support between coaches and athletes play an important role not only as social support but also in shaping how athletes perceive challenging situations in competitive and high-pressure contexts (Jang and Seong, 2017; Choi and Cho, 2019; Mageau and Vallerand, 2003; Jowett and Poczwardowski, 2007). In other words, when athletes maintain a good relationship with their coaches, they are more likely to view difficult tasks or training situations not merely as burdens or sources of stress, but as opportunities for growth and self-improvement.

Previous studies have emphasized that a positive coach–athlete relationship contributes to athletes' psychological stability, emotional support, and sense of belonging, all of which help athletes activate their self-efficacy and intrinsic motivation in challenging situations (Blascovich and Mendes, 2010; Deci and Ryan, 1985; Gustafsson et al., 2011). In this sense, the coach–athlete relationship provides an essential foundation that allows athletes to approach stressful situations with a challenge-oriented mindset, ready to utilize their psychological resources effectively. Moreover, when coaches provide appropriately challenging tasks while also offering emotional support and encouragement, athletes are more likely to perceive these tasks as positive and achievable goals rather than as threats or potential failures (Raedeke and Smith, 2001; Cresswell and Eklund, 2007). This process strengthens athletes' challenge appraisal, promotes the effective use of their psychological resources, and ultimately contributes to preventing burnout.

Overall, this study highlights that the coach–athlete relationship is a key social factor in fostering challenge appraisal. It suggests that building strong, positive relationships with athletes goes beyond relational satisfaction; it helps athletes perceive difficult situations positively and maintain their self-efficacy and intrinsic motivation. Therefore, strategies aimed at strengthening the coach–athlete relationship can be highly effective for enhancing challenge appraisal and preventing psychological burnout among adolescent athletes.

Second, the findings of this study showed that challenge appraisal had a significant negative effect on athlete burnout. This indicates that athletes who perceive stressful or demanding situations as challenges, rather than threats, are less likely to experience burnout. In other words, when athletes evaluate training sessions or competitions positively and feel capable of handling the demands, they can maintain their motivation and psychological stability even under high pressure (Blascovich and Tomaka, 1996; Blascovich and Mendes, 2010). Previous research has highlighted that challenge appraisal activates both psychological and physiological resources, helping athletes to cope with stress more effectively and to sustain intrinsic motivation (Deci and Ryan, 1985; Gustafsson et al., 2011). Athletes who approach demanding situations with a challenge-oriented mindset are more likely to interpret setbacks or failures as opportunities for growth rather than as personal threats. This positive evaluation promotes adaptive coping strategies, resilience, and self-efficacy, which collectively reduce the risk of burnout. Moreover, setting and pursuing challenging goals can enhance athletes' sense of competence and encourage them to develop their skills. Athletes with a high level of challenge appraisal tend to engage in continuous effort and self-directed practice, which not only strengthens their performance but also mitigates the emotional and physical exhaustion associated with burnout (Lonsdale et al., 2009; Raedeke and Smith, 2001).

Taken together, these results suggest that challenge appraisal functions as a key psychological factor in preventing athlete burnout. Encouraging athletes to perceive demanding tasks as achievable challenges can foster intrinsic motivation, strengthen coping abilities, and ultimately help maintain long-term psychological well-being in competitive sports.

Third, the study demonstrated that challenge appraisal partially mediates the relationship between the coach–athlete relationship and athlete burnout. This finding indicates that positive coach–athlete relationships can indirectly reduce burnout by fostering higher levels of challenge appraisal. Previous studies have shown that supportive and trusting relationships with coaches enhance athletes' psychological resources, such as confidence, emotional security, and perceived competence, which influence how athletes evaluate demanding situations (Jowett and Poczwardowski, 2007; Isoard-Gautheur et al., 2016). In other words, when athletes perceive their coaches as supportive and trustworthy, they are more likely to interpret demanding tasks as challenges rather than threats, and this positive appraisal helps protect them against burnout (Blascovich and Tomaka, 1996; Blascovich and Mendes, 2010). This mediating pathway suggests that interventions aimed at strengthening the coach–athlete relationship may be more effective when they also encourage athletes to view training and competition demands as meaningful and achievable challenges.

Overall, these findings suggest that the coach–athlete relationship and challenge appraisal work together to influence athlete burnout. A high-quality coach–athlete relationship provides emotional and social support that reduces perceived stress and promotes a sense of belonging and trust (Schellenberg et al., 2013; Isoard-Gautheur et al., 2016). At the same time, high levels of challenge appraisal allow athletes to maintain intrinsic motivation, cope more effectively with stress, and reduce the risk of burnout by activating adaptive psychological and physiological responses (Deci and Ryan, 1985; Gustafsson et al., 2011; Lonsdale et al., 2009). In practical terms, coaches who foster supportive relationships while providing appropriately challenging tasks can help athletes achieve both psychological stability and personal growth. This approach is particularly important for adolescent athletes, for whom social support and cognitive appraisals play a critical role in motivation and emotional regulation (Raedeke and Smith, 2001). Taken together, these results highlight the importance of addressing both social and psychological factors through an integrated approach when developing strategies to prevent athlete burnout.

### 5.1 Practical implications

This study underscores the coach's pivotal role in fostering athletes' psychological well-being. To enhance relationship quality, coaches should prioritize supportive communication, such as active listening and consistent encouragement, and adopt an empathetic approach to reduce athletes' perceived task difficulty. Furthermore, providing autonomy-supportive feedback can helps athletes interpret demanding training and competition situations as opportunities for growth rather than threats. For instance, coaches can frame mistakes as part of the learning process and collaboratively set achievable goals. Establishing a psychologically safe environment, in which athletes feel respected and can express their thoughts without fear of negative consequences, is also essential for promoting challenge appraisal. These strategies strengthen coping resources and intrinsic motivation, ultimately mitigating the risk of burnout. Consequently, coach education intervention programs should integrate these relational and cognitive components to support the long-term performance and psychological well-being of adolescent athletes.

## 6 Conclusions

The fundamental guiding principle of team sports is to prioritize the team over the individual and to share both success and failure as collective experiences. Within such environments, athletes grow through cooperation and competition, and effective teamwork serves as a core determinant of performance in team sports. To achieve this, team members must engage in sincere and respectful interactions, grounded in mutual understanding, active listening, and cooperation toward shared goals, both mentally and technically.

From this perspective, the present study examined how the coach–athlete relationship and challenge appraisal function in relation to athlete burnout within team sports. The findings indicated that both variables played important roles in reducing burnout among adolescent athletes. Athletes who perceived a higher-quality relationship with their coach tended to maintain greater emotional stability and sustained motivation, which contributed to lower levels of burnout. Likewise, athletes with higher levels of challenge appraisal were better able to cope with demanding training and competition situations, thereby reducing their vulnerability to burnout. A notable finding of this study was that the coach–athlete relationship exerted a significant positive direct effect on challenge appraisal. This finding suggests that a positive relationship with the coach contributes to athletes' challenge appraisal by providing emotional and motivational resources. At the same time, challenge appraisal also represents athletes' individual cognitive interpretations of sport-related demands. In this regard, challenge appraisal reflects an individual cognitive tendency shaped by both relational influences and athletes' internal psychological processes, such as their capacity to interpret and manage stressful demands.

Overall, the findings indicate that the coach-athlete relationship and challenge appraisal influence athlete burnout through partly independent psychological processes. These findings suggest that athlete burnout is influenced by multiple mechanisms involving both social-environmental and individual-level factors. Based on these findings, the prevention of burnout in adolescent athletes requires a balanced approach that addresses the quality of the coach–athlete relationship while simultaneously fostering a psychological climate that encourages athletes to perceive training and competition demands as challenges rather than threats. In practical terms, coaches should engage in supportive communication, demonstrate empathy, provide autonomy-supportive feedback, and create psychologically safe environments to enhance athletes' challenge appraisal and well-being.

However, the findings of this study should be interpreted with caution. First, the cross-sectional research design limits the ability to draw clear causal inferences among the variables. Second, the reliance on self-report measures may introduce common method bias and social desirability effects. Third, as the sample consisted of adolescent team-sport athletes within a specific cultural context, caution should be exercised when generalizing the findings to other populations. Future research is therefore encouraged to employ longitudinal or experimental designs to clarify causal relationships and to incorporate multiple data sources, such as coach ratings, peer assessments, or observational methods, to enhance the validity of the findings. Additionally, examining diverse age groups, sport types, and cultural settings, as well as exploring potential mediating or moderating variables such as resilience, motivational climate, or team cohesion, would provide a more comprehensive understanding of athlete burnout.

Despite these limitations, the present study contributes to the literature by demonstrating that the coach–athlete relationship and challenge appraisal operate as independent psychological pathways through which athlete burnout can be explained. In doing so, it extends theoretical understanding of burnout in team sports and offers meaningful practical implications for the development of more effective burnout prevention strategies in applied sport settings.

## Data Availability

The original contributions presented in the study are included in the article/supplementary material, further inquiries can be directed to the corresponding authors.
